# Supplementary material for: Utilization of government healthcare services by adult leprosy patients in the Western Province, Sri Lanka
Source: PLoS Negl Trop Dis. 2020 Dec 31;14(12):e0008973. doi: 10.1371/journal.pntd.0008973 (PMC7806162; doi:10.1371/journal.pntd.0008973)
Supplement: S1 Text — (DOCX) [file pntd.0008973.s001.docx]

**S1 Text**

**Utilization of healthcare services by leprosy patients**

Interview only Pausibacillary adult leprosy patients diagnosed at least 6 months and above and Multibacillary adult leprosy patients diagnosed at least 12 months and above for this section.

**PART l –Information on disease and treatment**

| 1 | Serial number ALC |  |
| --- | --- | --- |
| 2 | Clinic registration number |  |
| 3 | Name of the clinic |  |
| 4 | District |  |
| 5 | MOH area |  |
| 6 | Contact number |  |

Please mark (X) for the correct response.

7. Disease Classification -WHO

| 1 | Pausibacillary(PB) |  |
| --- | --- | --- |
| 2 | Multi bacillary(MB) |  |

Note: Refer section 2 in the guideline for classification

8. Date of starting MDT treatment

| D | D | M | M | Y | E | A | R |
| --- | --- | --- | --- | --- | --- | --- | --- |
|  |  |  |  |  |  |  |  |

9. Duration of drug treatment up to now in completed months

**PART ll – Socio Demographic Data**

10. Age in completed years

11. Sex

| 1 | Male |  |
| --- | --- | --- |
| 2 | Female |  |

12. Marital status

| 1. | Married |  |
| --- | --- | --- |
| 2. | Unmarried |  |
| 3. | Widowed |  |
| 4. | Divorced |  |
| 5. | Other(mention) |  |

13. Maximum educational level

| 1. | No schooling |  |
| --- | --- | --- |
| 2. | Up to Grade 5 |  |
| 3. | Up to Grade 8 |  |
| 4. | Up to Ordinary Level |  |
| 5. | Up to Advanced Level |  |
| 6. | Tertiary education |  |

14. Occupation

| 1 | Unemployed |  |
| --- | --- | --- |
| 2 | Self-employed |  |
| 3 | Paid employment |  |

15. If you are employed, name of your occupation?

Note: Refer section 2 in the guideline

16. Monthly Income

|  | Income | Individual | Family |
| --- | --- | --- | --- |
| 1. | Less than Rs 10000 |  |  |
| 2. | Rs 10001-20000 |  |  |
| 3. | Rs 20001- 30000 |  |  |
| 4. | Rs 30001-40000 |  |  |
| 5. | Rs 40001-50000 |  |  |
| 6. | Rs 50001-60000 |  |  |
| 7. | More than Rs >60001 |  |  |

**Part III**

**A . History of the disease**

17. What was your initial experience on this disease?(Mark “**X**” in the appropriate cage. Multiple responses possible.)

| 1 | Developed pale or reddish patches over the skin with loss or decreased sensation in the skin patch |  |
| --- | --- | --- |
| 2 | Developed numbness in hands and foot |  |
| 3 | Developed nodules over the skin |  |
| 4 | Developed deformity(Claw hand/ Foot drop/ Foot ulcer) |  |
| 5 | Any other( Please mention) |  |

18. Where did you go for initial consultation?

| 1 | General practitioner |  |
| --- | --- | --- |
| 2 | Government hospital |  |
| 3 | Consultant in the Private hospital |  |
| 4 | Ayurveda hospital (Government) |  |
| 5 | Ayurveda treatment Centre(Private) |  |

19. Who referred you to this center?

| 1 | Self-referral |  |
| --- | --- | --- |
| 2 | General practitioner |  |
| 3 | Government hospital clinic |  |
| 4 | Consultant in the private sector |  |
| 5 | By MOH office staff during the household leprosy inspection |  |
| 6 | School Medical Inspection |  |
| 7 | Contact screening by PHI |  |
| 8 | Any other(please mention) |  |

**B. Status of Clinic Utilization**

20. Date of registration to the clinic

| D | D | M | M | Y | E | A | R |  |
| --- | --- | --- | --- | --- | --- | --- | --- | --- |
|  |  |  |  |  |  |  |  |  |

21.Date of diagnosis

| D | D | M | M | Y | E | A | R |
| --- | --- | --- | --- | --- | --- | --- | --- |
|  |  |  |  |  |  |  |  |

22.Date of starting drug treatment

| D | D | M | M | Y | E | A | R |  |
| --- | --- | --- | --- | --- | --- | --- | --- | --- |
|  |  |  |  |  |  |  |  |  |

23. Duration of drug treatment up to now in completed months.

24. Place of diagnosis

| 1 | In this clinic |  |
| --- | --- | --- |
| 2 | Other clinic |  |

25. What is the time duration for the treatment

|  | **Event** | **Days** | **Months** |
| --- | --- | --- | --- |
| 1 | From the onset of symptoms to presentation to a doctor/health facility for first time (patient related delay ) |  |  |
| 2 | From presentation to a doctor (date of registration to the clinic) to diagnosis |  |  |
| 3 | From diagnosis to starting treatment |  |  |
| 4 | From presentation to starting treatment (Health care system delay) |  |  |
| 5 | From onset of symptoms to starting treatment (overall delay) |  |  |

26. Frequency of clinic attendance to get drug treatment, following registration?

| Months | 1 | 2 | 3 | 4 | 5 | 6 | 7 | 8 | 9 | 10 | 11 | 12 | Continued more than 12 month |
| --- | --- | --- | --- | --- | --- | --- | --- | --- | --- | --- | --- | --- | --- |
| Date of clinic attendance |  |  |  |  |  |  |  |  |  |  |  |  |  |

27. Number of clinic visits to get drug treatment

| 1 | Total number of clinic visits attended |  |
| --- | --- | --- |
| 2 | Required number to be attended |  |

28.Were you hospitalized during the course of treatment due to an event related to leprosy ?

| 1 | Yes |  |
| --- | --- | --- |
| 2 | No |  |

29 If Yes,

|  | Name of the Hospital | Reason for admission | No of days spent in the Hospital |
| --- | --- | --- | --- |
| 1 |  |  |  |
| 2 |  |  |  |
| 3 |  |  |  |

30.Are you attending to the leprosy clinic nearest to your current residence ?

| 1 | Yes |  |
| --- | --- | --- |
| 2 | No |  |

31. If answer is No, reason for not attending to nearest clinic?

| 1 | To prevent people known to me coming to know my illness |  |
| --- | --- | --- |
| 2 | Lack of laboratory facilities |  |
| 3 | Poorly maintained premises |  |
| 4 | Lack of friendliness by the health care workers |  |
| 5 | Over crowded |  |
| 6 | To continue the treatment with the specialist who was treated from the beginning |  |
| 7 | Believing that this hospital provide better service |  |
| 8 | Other reason(please specify) |  |

**C. Knowledge of the patient**

32. Leprosy is more prone to develop in

|  |  | Correct | Incorrect | Don’t know |
| --- | --- | --- | --- | --- |
| 1 | People with malnutrition |  |  |  |
| 2 | People with poverty |  |  |  |
| 3 | People living in the overcrowded houses |  |  |  |
| 4 | Poor personal hygiene |  |  |  |

33. Leprosy can be transmitted by

|  |  | Correct | Incorrect | Don’t know |
| --- | --- | --- | --- | --- |
| 1 | Nasal droplets of a affected person |  |  |  |
| 2 | Using same toilet |  |  |  |
| 3 | Bath in the same well |  |  |  |
| 4 | Closely live with a affected patient who is not on treatment |  |  |  |
| 5 | Genetically |  |  |  |
| 6 | Live with a patient who is on treatment |  |  |  |

34. Leprosy patient can present with

|  |  | Correct | Incorrect | Don’t know |
| --- | --- | --- | --- | --- |
| 1 | Hypo pigmented anesthetic patches |  |  |  |
| 2 | Nodules over the skin |  |  |  |
| 3 | Cough |  |  |  |
| 4 | Muscle weakness |  |  |  |
| 5 | Disability |  |  |  |

35. Can a leprosy patient attend to social activities while on treatment?

| 1 | Correct |  |
| --- | --- | --- |
| 2 | Incorrect |  |
| 3 | Don’t know |  |

Find the correct statement regarding Leprosy. Mark “**X**” in the appropriate cage.

|  | Condition | Correct | Incorrect | Don’t know |
| --- | --- | --- | --- | --- |
| 36 | Leprosy leads to vision impairment in some patients. |  |  |  |
| 37 | Leprosy does not affect to nerve function. |  |  |  |
| 38 | Leprosy leads to disability and disfigurement if left untreated. |  |  |  |
| 39 | Leprosy can be completely cured by taking regular treatment. |  |  |  |

**Part 1V- Utilization of Field Services**

**D. Knowledge of the patient on field services**

40. Can the MOH office in your area provide services for leprosy affected families?

| 1 | Yes |  |
| --- | --- | --- |
| 2 | No |  |
| 3 | Not relevant |  |

41. Does PHI have a role to examine your family members?

| 1 | Yes |  |
| --- | --- | --- |
| 2 | No |  |

42. Do you know the location of your MOH office?

| 1 | Yes |  |
| --- | --- | --- |
| 2 | No |  |
| 3 | Not relevant |  |

**E. Provision of Field Health Services**

43. Once you have diagnosed, did the PHI visit to your house?

| 1 | Yes |  |
| --- | --- | --- |
| 2 | No |  |
| 3 | No, but contact over the phone and gave necessary advises. |  |

If the answer is yes,

44. Did he give health education to you?

| 1 | Yes |  |
| --- | --- | --- |
| 2 | No |  |

45. Did the PHI carry out screening of your male family members for leprosy?

| 1 | Yes |  |
| --- | --- | --- |
| 2 | No |  |

46. If the response is No, from where the family screening was done

| 1 | Hospital |  |
| --- | --- | --- |
| 2 | MOH office |  |
| 3 | Family member screening was not done |  |

47. Did all your family members undergo leprosy screening?

| 1 | Yes |  |
| --- | --- | --- |
| 2 | No |  |

48. If all your family contacts not examined, reason for that?

| 1 | PHIs are not supposed to examine female contacts. |  |
| --- | --- | --- |
| 2 | Lack of time to go to the clinic/MOH office. |  |
| 3 | Not advised to get family members examined. |  |
| 4 | Financial problems affect to health care visit. |  |
| 5 | Not aware that leprosy can be spread from one person to other. |  |
| 6 | Family members not had symptoms suggestive of leprosy |  |
| 7 | Temporary living alone. |  |
| 8 | Illness of family member causing delay to attend to hospital |  |
| 9 | Conceal the disease condition from family members |  |
| 10 | Any other(please mention) |  |

**F. Patient’s perception on field services**

49. Would you prefer the visits of health care workers to your house?

| 1 | Yes |  |
| --- | --- | --- |
| 2 | No |  |

50. If not, the reason for that?

| 1 | To avoid neibours knowing the disease condition |  |
| --- | --- | --- |
| 2 | They may create unnecessary fear among family members. |  |
| 3 | Any other(please mention) |  |
