# Supplementary material for: Utilization of government healthcare services by adult leprosy patients in the Western Province, Sri Lanka
Source: PLoS Negl Trop Dis. 2020 Dec 31;14(12):e0008973. doi: 10.1371/journal.pntd.0008973 (PMC7806162; doi:10.1371/journal.pntd.0008973)
Supplement: Attachment — Submitted filename: Review of Liyanage, N. 2020 manuscript.pdf [file pntd.0008973.s004.pdf]

In this manuscript (PNTD-D-20-01458), the authors performed a descriptive cross-sectional study of the Western Province of Sri Lanka to assess utilization of available healthcare resources by diagnosed leprosy patients. It was an interesting study that found that within this population, there is a considerable delay between when a patient initially notices leprosy symptoms and actually seeks medical treatment. Compared to similar studies in both China and Brazil, these authors demonstrated that their patient population accesses the Sri Lankan healthcare services faster than their Chinese or Brazilian counterparts. While this was an interesting study, there are some minor modifications needed prior to publication.

Author Summary and Introduction:

- The second half of the author summary reads awkwardly. I understand what the authors are trying to say, but I think some minor rewording or sentence restructuring would greatly improve the clarity in the summary.
- The authors thoroughly highlight their primary objective of measuring utilization of the healthcare system throughout both the author summary and introduction. However, there isn't any mention of their secondary objective of determining the levels of leprosy knowledge among their population. A brief mention of this objective and how it could play a role in how effectively patients access the healthcare system would be useful in these sections.

Methods:

- It would be helpful for the authors to give a brief background on the Western Province. In particular, is it primarily urban or rural, since this could greatly impact patient-related delay in seeking medical care.
- I was unsure what was meant by "ongoing one year period" when the authors were describing service utilization in a clinical setting.
- Also, some brief clarification on the sampling used would be beneficial. It was unclear the role played by the clinical leprosy register in recruiting patients and how that differed from other recruitment strategies.

Results:

- Change "Type 11" to Type 2.
- Check that all table headings and labels are consistent among all tables.

Discussion and Conclusion:

- The authors need to discuss the limitations of their work in their discussion section. Were any biases introduced?
- The acronym OPDs is used in the Discussion, and I don't think it was spelled out earlier in the text, so please correct that.

- At the end of the first paragraph in the discussion, the authors state that the PHI is only able to examine males. This is the first time this limitation is mentioned, and it wasn't explained why this occurred.
- I found the authors' comparison of their results to other countries' results to be very interesting and informative. However, I think that the authors could flesh out this section a bit more by discussing why their results are better or worse than the other results. Is there a particular program in Sri Lanka that isn't present in China for instance? I think a more thorough discussion of these points would help to better demonstrate the relevance of their study both for Sri Lanka and for the larger leprosy community, which is unfortunately lacking in the present draft.
- Finally, in their conclusions, the final paragraph with the authors' recommendations was a bit vague. More detail in this final section possibly based off of the above comment about the discussion would be helpful.
