# Supplementary material for: Utilization of government healthcare services by adult leprosy patients in the Western Province, Sri Lanka
Source: PLoS Negl Trop Dis. 2020 Dec 31;14(12):e0008973. doi: 10.1371/journal.pntd.0008973 (PMC7806162; doi:10.1371/journal.pntd.0008973)
Supplement: Attachment — Submitted filename: PLOS review.docx [file pntd.0008973.s003.docx]

**Author summary has been modified by the reviewer**

Leprosy is a chronic progressive bacterial infection caused by *Mycobacterium Leprae*. It mainly attacks the skin, peripheral nerves in the hands, feet and eyes, causing numbness or weakness of

the affected area and resulting in chronic morbidities such as vision impairment, limb disability, trophic ulcers and nerve involvement. Leprosy is assumed to be spread via the respiratory

system through nasal droplets. It is commonly found among people living in poor socioeconomic

conditions. Therefore to prevent getting complications, early diagnosis and

treatment is mandatory. Our aim was to identify any delays in diagnosis and treatment and whether patients are utilizing the existing facilities. Knowledge of the healthcare workers to identify the disease and patients’ knowledge to

continue the treatment is of equal importance to reduce the case load and prevent developing

complications. To find the reasons why the services are not utilized and communicate the true

picture to the decision makers are the objectives of this study.

**Introduction**

Health care utilization is the measure of the population’s use of the health care services available

to them [1,2]. Health care utilization and health status are indicators to measure how efficiently a

health care system provides services in a population. Service utilization is the extent to which

people are making use of the services that already exist in the community or at an organization

[3,4,5]. The importance of assessing utilization of a health service is that the policymakers will

be aware of the existing services and underutilized services which will help them to decide

which services should be expanded or which should probably be discontinued. It

will also assist health care providers to plan future programs and use their resources more

effectively. Assessing service utilization is beneficial since it can be monitored and followed for

any changes over time [6,7].

**Reviewer comments**

- Suggested grammar changes
  - Page 12 line 2 “were resided in the Colombo district” please omit “were”
  - Page 12 line 4 “were attended to the CLC (n=290, 43.2%)” **attended the CLC**
  - Page 12 “A majority of the study sample consisted of” the majority
  - Page 12 “ordinary level” ordinary level examination
- Monthly income is given in LKR. It may appear more meaningful to an international viewership if the USD equivalent is given
- In page 15 it is mentioned “The most common reason for hospital admission was type 11 reaction with fever and rash (n=24, 26%) and to diagnose leprosy (n=24, 26%). The majority were hospitalized for less than 7 days (n=62, 67.4%)” . The statement that 24 patients were admitted for the diagnosis seems unlikely since leprosy is a disease that can be diagnosed and managed on out patient basis. Can you please give more details of the admissions “for diagnosis”?
- Page 17 “PHI visited their houses and gave health education (n=363, 54%) by PHI” delete **by PHI**
- Among the patients, 482 (71.7%) **preferred** a home visit by health care workers. Consented to would be more appropriate than preferred a home visit.
- More than half of the sample (n=351, 52.2%) **said,** leprosy cannot be transmitted by living with a person who is on treatment. **knew that** would be more appropriate
