# Supplementary material for: Utilization of government healthcare services by adult leprosy patients in the Western Province, Sri Lanka
Source: PLoS Negl Trop Dis. 2020 Dec 31;14(12):e0008973. doi: 10.1371/journal.pntd.0008973 (PMC7806162; doi:10.1371/journal.pntd.0008973)
Supplement: Attachment — Submitted filename: 2020 11 01 Correction sheet.docx [file pntd.0008973.s005.docx]

**Title*: Utilization of Government Healthcare Services by Adult Leprosy Patients in the Western Province, Sri Lanka***

***PNTD-D-20-01458***

**Correction sheet made for the reviewer comments**

|  | **Section** | **Details of the reviewer comments** | **Description of the changes made to the manuscript** |
| --- | --- | --- | --- |
| 1 | **Abstract** |  |  |
| 1.1 | P2,3 | Reviewer 2 :Discuss on Leprosy knowledge | Corrections done. |
| 2 | **Author summary** |  |  |
| 2.1 | P3 | Reviewer 1:Not clearly presented | Corrections done. |
| 2.2 | P3 | Reviewer 2:Reword the Author summary, Discuss on Leprosy knowledge | Corrections done. |
| 3 | **Introduction** |  |  |
| 3.1 | P4 | Reviewer 2 :Discuss on Leprosy knowledge | Corrections done. |
| 4 | **Methods** |  |  |
| 4.1 | P 6 | Reviewer 2:  Explanation on ongoing one year period | The Correction was done in the text. Further to that an explanation is given below,  Since the study is a descriptive cross-sectional in nature, we assessed patient clinic attendance % during their treatment period.  Therefore, time period for MB patient was considered as one year and above; for PB patient 6 months and above, following diagnosis.  Clinic attendance percentage (clinic utilization) was calculated retrospectively using their clinic records as follows,  Clinic utilization= (actual no of clinic visit attended by patients) / (Total number of due visits for a particular treatment period). |
| 4.2 | P-6 | Reviewer 2:  Give a brief background on Western Province | Corrections done. |
| 4.3 | P-6 | Reviewer 2:  Slightly unclear about the role of clinic leprosy register | Corrections done. |
| 5 | **Results** |  |  |
| 5.1 | P12 | Reviewer 2:”Type 11 reaction” should be changed as “Type2 reaction” | Corrections done. |
| 5.2 | P12 | Reviewer 1?Why admitted for diagnosis | We do agree that leprosy can be diagnosed at OPD level (for majority of patients).  But there are a group of patients who are attending clinics who need to be admitted due to  -Being very ill  -With doubtful diagnosis  -Vulnerable patients (drug addicts, prisoners etc.)  -Presenting with severe reactions for the first time (or subsequently)  These special cases are being admitted to a ward by the Consultant Dermatologist and while managing the other symptoms they do SSS test, skin biopsy, and other relevant investigations. |
| 6 | **Discussion** |  |  |
| 6.1 | P17,18,19,20 | Reviewer 2: Expand the discussion section | Corrections done. |
|  | P17 | Explain abbreviation of OPD | Corrections done. |
|  | P17 | Explain the limitation of why PHI is able to examine males, | Corrections done. |
|  | P19 | Compare your study results with leprosy campaign results | Corrections were done with the available data. No previous studies were conducted in the field of service utilization in Sri Lanka related to leprosy (No studies were conducted by the Anti Leprosy Campaign as well) |
| 6.2 | P 20 | Reviewer 2: Discuss limitations of your own study, were any biases introduced? | Corrections done. |
| 7 | **Conclusions** |  |  |
| 7.1 | P20,21 | Reviewer 2: Expand the conclusions both to Sri Lanka and to the Global leprosy field, revise the last paragraph after amending the discussion section | Corrections done. |
